# The Hemagglutinin of Bat-Associated Influenza Viruses Is Activated by TMPRSS2 for pH-Dependent Entry into Bat but Not Human Cells

**DOI:** 10.1371/journal.pone.0152134

**Published:** 2016-03-30

**Authors:** Markus Hoffmann, Nadine Krüger, Pawel Zmora, Florian Wrensch, Georg Herrler, Stefan Pöhlmann

**Affiliations:** 1 Infection Biology Unit, German Primate Center, Göttingen, Germany; 2 Institute of Virology, University of Veterinary Medicine Hannover, Hannover, Germany; Centre of Influenza Research, The University of Hong Kong, HONG KONG

## Abstract

New World bats have recently been discovered to harbor influenza A virus (FLUAV)-related viruses, termed bat-associated influenza A-like viruses (batFLUAV). The internal proteins of batFLUAV are functional in mammalian cells. In contrast, no biological functionality could be demonstrated for the surface proteins, hemagglutinin (HA)-like (HAL) and neuraminidase (NA)-like (NAL), and these proteins need to be replaced by their human counterparts to allow spread of batFLUAV in human cells. Here, we employed rhabdoviral vectors to study the role of HAL and NAL in viral entry. Vectors pseudotyped with batFLUAV-HAL and -NAL were able to enter bat cells but not cells from other mammalian species. Host cell entry was mediated by HAL and was dependent on prior proteolytic activation of HAL and endosomal low pH. In contrast, sialic acids were dispensable for HAL-driven entry. Finally, the type II transmembrane serine protease TMPRSS2 was able to activate HAL for cell entry indicating that batFLUAV can utilize human proteases for HAL activation. Collectively, these results identify viral and cellular factors governing host cell entry driven by batFLUAV surface proteins. They suggest that the absence of a functional receptor precludes entry of batFLUAV into human cells while other prerequisites for entry, HAL activation and protonation, are met in target cells of human origin.

## Introduction

Influenza A viruses (FLUAV) are enveloped, negative stranded RNA viruses that pose a major threat to public health [1]. The ability of FLUAV to constantly adapt to immune pressure allows these viruses to continuously circulate in the human population, resulting in annual influenza epidemics (seasonal influenza [2, 3]). Infants, children and the elderly are at particular risk of developing severe disease upon infection with seasonal FLUAV and it has been estimated that world-wide 250,000 to 500,000 people die each year of seasonal influenza [1]. Waterfowl has been shown to constitute the natural reservoir of FLUAV [4, 5] from which viruses with pandemic potential can be directly transmitted to humans or can emerge upon reassortment of avian and human FLUAV [2, 4, 6]. Influenza pandemics might have dramatic consequences, as highlighted by the 30–50 million deaths attributed to the influenza pandemic of the years 1918/1919 (Spanish influenza [7, 8]).

The viral surface proteins hemagglutinin (HA) and neuraminidase (NA) facilitate FLUAV entry and release from target cells, respectively. HA facilitates viral attachment to cells by binding to sialic acids on cell surface proteins or lipids [9–11] and, upon proteolytic activation by a host cell protease and exposure to endosomal low pH, mediates fusion of the viral membrane with the endosomal membrane [12–14]. In contrast, NA promotes release of progeny particles from infected cells by removing sialic acids from cell surface factors. Based on sequence and antigenic properties, sixteen HA (H1-16) and nine NA (N1-9) subtypes have been identified, and viruses representing all HA and NA subtypes are circulating in waterfowl [4, 15]. However, FLUAV-related viruses were recently discovered in New World bats [16, 17], provisionally termed bat-associated influenza A-like viruses (batFLUAV), and were shown to harbor HA- and NA-like proteins (termed HAL and NAL), which constitute new subtypes, H17/H18 (HL17/HL18) and N10/N11 (NL10/NL11), respectively. The question whether these viruses have the potential to infect and spread in humans is the focus of current research efforts.

Attempts to isolate batFLUAV were unsuccessful [16, 17] but, employing reverse genetics, it was demonstrated that the viral replication machinery and interferon antagonists are functional in mammalian cells [16, 18–21]. In contrast, the HAL and NAL proteins of batFLUAV were incompatible with viral spread in the cell culture systems examined so far [18, 19] for at present unknown reasons. Biochemical and structural studies imply that batFLUAV-HAL, unlike FLUAV-HA, does not engage sialic acids (SA) for host cell entry [17, 22–24], and that batFLUAV-NAL, unlike FLUAV-NA, neither shows neuraminidase activity nor possesses an active site that would allow interaction with sialic acids [17, 25–27]. However, it is currently unclear whether HAL and NAL can facilitate viral entry into certain target cells and it is unknown which determinants control the entry process.

Here, we utilize rhabdoviral vectors to analyze host cell entry driven by batFLUAV-HAL and -NAL. We show that HAL facilitates entry into certain bat but not human cell lines and that entry is independent of sialic acids. In contrast, HAL-driven entry was dependent on prior proteolytic activation of HAL and endosomal acidification. Moreover, we provide evidence that HAL can utilize the cellular protease TMPRSS2 for its activation, suggesting that batFLUAV entry into human cells is mainly restricted at the stage of receptor engagement while proteolytic activation and triggering of HAL are not limiting the entry process.

## Materials and Methods

### Cell culture

The following cell lines were used as targets for transduction and expression experiments and were maintained in Dulbecco's modified Eagle's medium (PAA Laboratories), supplemented with 10% fetal bovine serum (Biochrom) and antibiotics (penicillin/streptomycin, PAA Laboratories): HEK-293T, Huh7, Vero, MDCK, BHK-21, as well as chiropteran cell lines from five different bat species, RoNi/7, HypNi/1.1, EidNi/41, EpoNi/22.1 and CpKd (Table 1). All non-bat-derived cell lines were obtained from collaborators. The fruit bat cell lines (RoNi/7, HypNi/1.1, EidNi/41, EpoNi/22.1) were a kind gift of C. Drosten and M. A. Müller and have been described previously [28–31]. The CpKd cell line was described elsewhere [28]. All cell lines were grown in a humidified atmosphere at 37°C and 5% CO_2_. For passaging and seeding, cells were detached by either resuspension in fresh culture medium (HEK-293T cells) or by the use of trypsin/EDTA (PAA Laboratories).

**Table 1:** Cell lines used to study batFLUAV tropism.

Name	Species	Organ
HEK-293T	Human (*Homo sapiens*)	Kidney
Huh7	Human (*Homo sapiens*)	Liver
Vero	African green monkey (*Chlorocebus aethiops*)	Kidney
MDCK	Dog (*Canis lupus familaris*)	Kidney
RoNi/7	Egyptian fruit bat (*Rousettus aegyptiacus*)	Kidney
HypNi/1.1	Hammer-headed fruit bat (*Hypsignathus monstrosus*)	Kidney
EidNi/41	Straw-colored fruit bat (*Eidolon helvum*)	Kidney
EpoNi/22.1	Buettikofer's epauletted fruit bat (*Epomops buettikoferi*)	Kidney
CpKd	Seba’s short-tailed bat (*Carollia perspicillata*)	Kidney

### Plasmids

Shuttle vectors harboring codon-optimized (for expression in human cells) open reading frames coding for the published amino acid (aa) sequences of the HAL of the two batFLUAV, A/little yellow-shouldered bat/Guatemala/153/2009 (H17/N10) (GenBank: CY103876.1, HAL17) and A/flat-faced bat/Peru/033/2010 (H18/N11) (GenBank: CY125945.1, HAL18), were purchased from a commercial service (Eurofins MWG Operon) and cloned into the pCG1 expression vector, that was kindly provided by R. Cattaneo, via *BamH*I and *Xba*I restriction sites. The pCAGGS-based expression plasmid for NAL of batFLUAV A/little yellow-shouldered bat/Guatemala/153/2009 (H17/N10) (GenBank: CY103878.1, NAL10) was provided by M. Schwemmle. HAL equipped with a C-terminal FLAG epitope (DYKDDDDK, HAL17-FLAG and HAL18-FLAG) were constructed by PCR and controlled for sequence integrity by automated sequence analysis. In addition, we used pCAGGS-based expression plasmids for the HA and NA of A/WSN/33 (H1N1) and A/Singapore/1/57 (H2N2) (GenBank: AY209895.1) [32, 33]. Furthermore, we employed pCAGGS-based expression plasmids for the HA of A/South Carolina/1/18 (H1N1) (GenBank: AF117241.1) and the NA of A/Brevig Mission/1/1918 (H1N1) (GenBank: AF250356.2) that were generated from previously used constructs [32]. The expression plasmid for the glycoprotein (G) of vesicular stomatitis virus (VSV, Indiana strain, VSV-G; GenBank: AJ318514.1) was generated by inserting the VSV-G ORF into the pCG1 expression vector and has been used in previous studies [28, 31, 34, 35]. Furthermore, expression plasmids for Nipah virus fusion protein (F, GenBank: AF212302) and glycoprotein (G, synthetic, GenBank: AF212302; derived from NiV/MY/HU/1999/CDC) were used [36]. For experiments analyzing proteolytic activation of HAL17 and HAL18 by human type-II transmembrane serine proteases (TTSPs), we employed expression plasmids for TMPRSS2, TMPRSS11E (DESC-1) and TMPRSS13 (MSPL), which have been described previously [32, 33].

### Production of rhabdoviral pseudotypes

We employed a replication-deficient VSV vector for pseudotyping that contains two separate open reading frames, coding for enhanced green fluorescent protein (EGFP) and firefly luciferase (fLuc), instead of the genetic information for VSV-G, VSV*ΔG-fLuc [28, 31, 35]. Propagation of VSV*ΔG-fLuc was performed in a previously described VSV-G-expressing, transgenic cell line [37]. Generation of VSV pseudotypes (VSVpp) was performed as follows: HEK-293T cells were transfected by calcium-phosphate precipitation with expression plasmids encoding viral surface proteins, VSV-G (positive control), NiV-F/G, FLUAV-HA and/or NA and batFLUAV-HAL and/or NAL, or empty plasmid (pCAGGS) as negative control. In order to investigate the potential of human TTSPs to proteolytically activate batFLUAV-HAL for host cell entry, we additionally cotransfected the cells with expression plasmids for TMPRSS2, DESC-1 or MSPL. At 16 h post transfection, the cells were inoculated with VSV*ΔG-fLuc at a multiplicity of infection of 3 for 1 h at 37°C and 5% CO_2_. Subsequently, the cells were washed and incubated with an anti-VSV serum to neutralize residual input virus. Finally, the cells received fresh culture medium and were further incubated for 16–20 h, before the VSVpp-containing supernatants were collected, clarified from cell debris by centrifugation and aliquoted. Aliquots were stored at 4°C for a maximum of 7 days.

For proteolytic activation of HA/HAL by trypsin, pseudotypes were incubated with bovine trypsin (Sigma-Aldrich; final concentration: 50 μg/ml) for 20 min at 37°C. Subsequently, trypsin was inactivated by addition of soybean trypsin inhibitor (Sigma-Aldrich; final concentration: 50 μg/ml).

### Treatment of cell lines with neuraminidase and ammonium chloride

To investigate the roles of sialic acids and endosomal acidification in batFLUAV entry, we used recombinantly-produced, bacterial sialidase (*Clostridium welchii*, Sigma-Aldrich) and ammonium chloride (Sigma-Aldrich). For treatment, the cell culture supernatant was removed and the cells were washed with phosphate buffered saline (PBS) before culture medium containing water (negative control), ammonium chloride (50 mM) or different concentrations of bacterial sialidase (1.5, 15 or 150 mU) was added. After 2 h of incubation at 37°C and 5% CO_2_, the supernatant was removed, the cells washed and then inoculated with pseudotypes, as described below.

### Transduction of cell lines with rhabdoviral pseudotypes and quantification of fLuc activity

All transduction experiments were performed in 96-well plates using quadruplicate samples. At 24 h post seeding, the cell culture medium was removed and the cells were washed with PBS. The cells were either directly inoculated with VSVpp or treated as specified above and then inoculated. VSVpp inoculation was performed for 1 h at 37°C and 5% CO_2_. Afterwards, the inoculum was removed and the cells were again washed and incubated with fresh culture medium for 16–18 h at 37°C and 5% CO_2_. For the quantification of the fLuc activity as an indicator of transduction efficiency, the cell culture supernatant was removed and the cells were washed with PBS. Next, 50 μl of 1x Luciferase Cell Culture Lysis Reagent (Promega) in PBS was added to each well and incubated for 30 min at room temperature, before the cell lysate was transferred to a white, opaque-walled 96-well plate (Thermo Scientific). The measurement of the fLuc activity was carried out in a microplate reader, Plate CHAMELEON (Hidex), using the MicroWin2000 software (version 4.44, Mikrotek Laborsysteme GmbH) and fLuc substrates from the Luciferase Assay System (Promega) or Beetle-Juice (PJK) kits. Transduction efficiency, represented by fLuc activity, was either displayed in counts per second (cps) or as normalized values.

### Immunofluorescence analysis of HAL expression

To assess expression of HAL proteins, we transfected BHK-21 cells that were grown on coverslips with expression plasmids for HAL17-FLAG or HAL18-FLAG using the Lipofectamine2000 reagent (ThermoFisher Scientific) according to manufacturers’ protocol. Cells transfected with an empty expression vector served as negative control. At 24 h post transfection, cells were fixed with 4% paraformaldehyde/PBS, permeabilized by incubation with 0.2 M Triton X-100/PBS (10 min at room temperature) and subsequently incubated with anti-FLAG (mouse, 1:1,000, Sigma-Aldrich) and Cy3-labeled anti-mouse (1:750, Sigma-Aldrich) antibodies. After each antibody incubation, the cells were washed three times with PBS and finally incubated with DAPI (Roth, 5 min/37°C) to stain the nuclei before the coverslips were mounted on glass slides using Mowiol (AppliChem) supplemented with anti-bleaching reagent (DABCO, Roth). Representative pictures were taken at a 10x magnification using a Nikon Eclipse Ti fluorescence microscope and the NIS Elements AR software (Nikon).

### Preparation of cell lysates and rhabdoviral pseudotypes for Western blot analysis

BatFLUAV-HAL cleavage was investigated by cotransfection of HEK-293T cells, which were grown in 6-well plates, with expression plasmids for batFLUAV-HAL and different TTSPs (TMPRSS2, DESC-1 or MSPL) or by incubation of HAL-expressing cells with trypsin (1, 5, 10, 50 μg/ml; 20 min/37°C) directly before cell lysates were produced. Cells cotransfected with empty plasmid and not subjected to trypsin treatment served as negative control. The 1918-HA served as positive control, since cleavage by TTSPs and trypsin has been previously shown [33]. At 24 h post transfection, the cells were washed with PBS, resuspended in 100 μl 2x SDS-containing lysis buffer (50 mM Tris [pH 6.8], 10% glycerol, 2% SDS, 5% β-mercaptoethanol, 0.1% bromophenol blue, 1 mM EDTA) and boiled for 20 min at 96°C. To assess incorporation of HAL into VSVpp, 1 ml of the respective pseudotypes was loaded onto a 20% sucrose cushion in PBS and centrifuged at 17,000x g for 2 h at 4°C. After discarding the supernatant, the pelleted pseudotypes were mixed with 30 μl 2x SDS-containing lysis buffer and boiled for 20 min at 96°C. Finally, all samples were subjected to immunoblot analysis. For this, anti-FLAG (mouse, 1:1,000, Sigma-Aldrich), anti-FLUAV (goat, 1:1,000, Millipore), anti-VSV-M (mouse, 1:1,000, Kerafast), anti-VSV-G (I1, mouse hybridoma supernatant from CRL-2700, ATCC, 1:200) and anti-ß-actin (mouse, 1:1,000, Sigma-Aldrich) served as primary antibodies. Peroxidase-coupled anti-mouse (1:10,000, Dianova) and anti-goat (1:5,000, Dianova) antibodies served as secondary antibodies. Signal detection was carried out in a ChemoCam imager together with the ChemoStar professional software (both Intas) using a self-made chemiluminescence substrate (recipe available upon request).

### Statistical analysis

In order to assess statistical significance, two-tailed student’s t-tests were performed.

## Results

### Both HAL17 and HAL18 are comparably expressed in mammalian cells and incorporated into rhabdoviral pseudotypes

**Fig 1:**
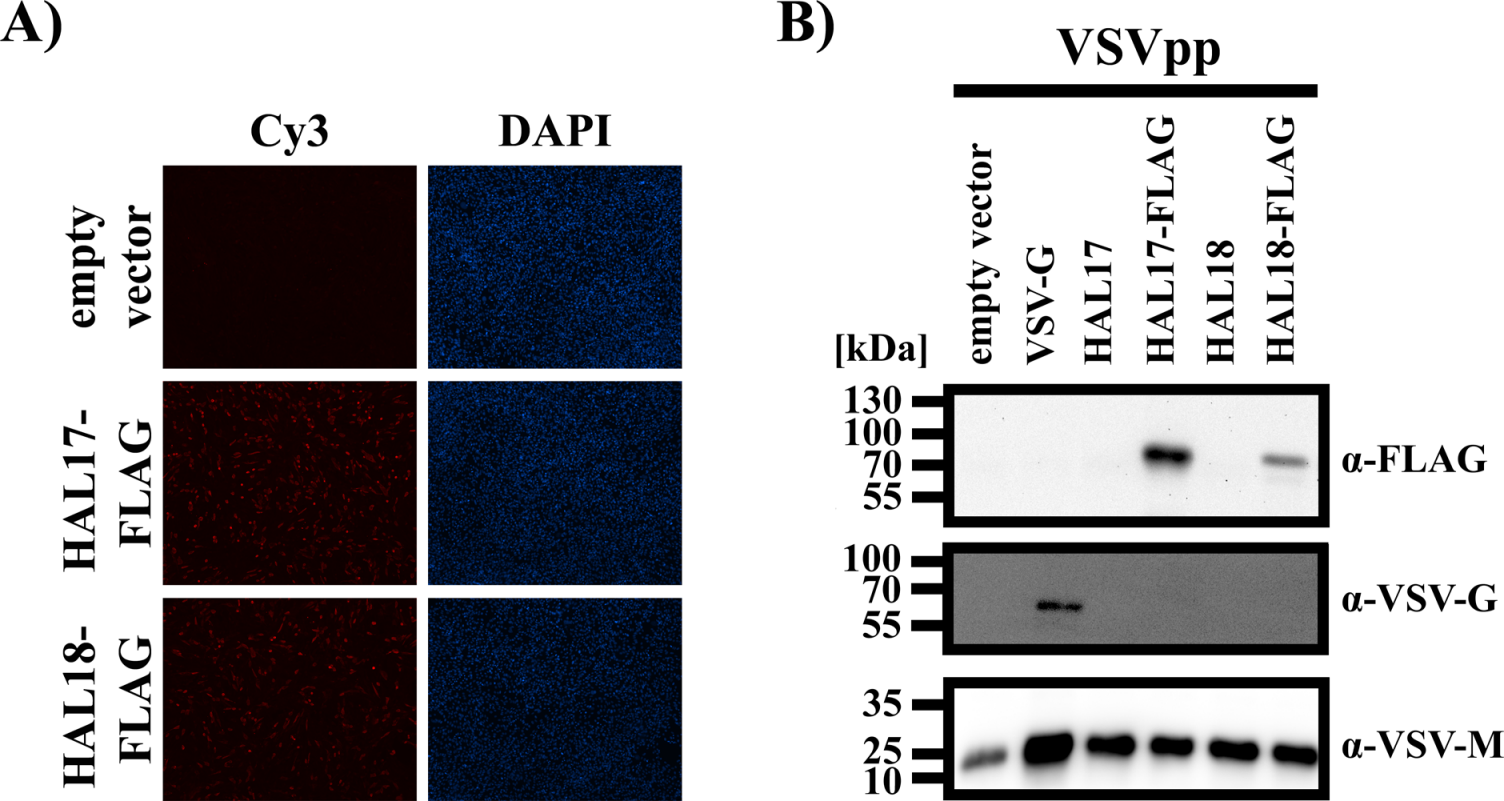
HAL of batFLUAV are robustly expressed and incorporated into rhabdoviral particles. (A) BHK-21 cells were transfected with the indicated HAL proteins harboring a C-terminal FLAG antigenic tag and protein expression was analyzed by immunofluorescence microscopy of permeabilized cells (magnification, 10x). Cells transfected with an empty expression vector served as negative control. Nuclei were visualized by DAPI staining. Similar results were obtained in a separate experiment. (B) For analysis of HAL incorporation into rhabdoviral particles, vesicular stomatitis virus (VSV)-based pseudotypes were pelleted though a 20% sucrose cushion and analyzed by SDS-PAGE and Western blotting with antibodies against the FLAG tag (α-FLAG), VSV glycoprotein (α-VSV-G) and matrix protein (α-VSV-M). The numbers on the left side of the blots indicate the molecular weight in kilo Daltons (kDa). The results were confirmed in an independent experiment.

To test whether HAL17 and HAL18 are comparably expressed and incorporated into rhabdoviral pseudotypes, both proteins were equipped with a C-terminal FLAG epitope (HAL17-FLAG, HAL18-FLAG), since no batFLUAV-HAL-specific antibody was available. Upon transfection of BHK-21 cells similar numbers of HAL-expressing cells were detected by fluorescence microscopy (Fig 1A) and the intensity of the fluorescence signals emitted by the cells was comparable, indicating robust expression of both batFLUAV-HAL proteins. In order to assess HAL incorporation into rhabdoviral pseudotypes, we pelleted pseudotype preparations through a sucrose cushion and subjected the samples to SDS-PAGE and immunoblotting. Using antibodies specific for the FLAG epitope, VSV-G and VSV matrix protein (VSV-M), we found that VSV-G, as expected, as well as both HAL17 and HAL18 proteins were incorporated into particles, with incorporation of HAL17 being more efficient than that of HAL18. (Fig 1B). Thus, both HAL17 and HAL18 were robustly expressed and incorporated into VSVpp, allowing their functional characterization.

### HAL of batFLUAV mediates cellular entry into bat cell lines and entry depends on proteolytic activation

It has been previously reported that HAL and NAL of batFLUAV are not compatible with viral spread in the cell lines tested so far [18, 19], suggesting that these proteins mediate entry into a restricted set of target cells or are even inactive. In order to gain insights into the functional activity of batFLUAV-HAL and NAL, we employed rhabdoviral vectors pseudotyped with these proteins. We inoculated cell lines from different host species, including those standardly used for FLUAV research, as well as cell lines derived from different bat species (Table 1), as they are the natural reservoir for batFLUAV. We chose bat cell lines known to be susceptible to infection by viruses of different families [28, 29, 31] and previously used to functionally characterize surface glycoproteins of bat-borne viruses [28, 36, 38]. It is well established that FLUAV-HA depends on proteolytic cleavage by host cell proteases to transit into an active form [12] and it has been previously reported that batFLUAV can be activated by exogenous trypsin [39–41]. Therefore, we assessed whether trypsin treatment of the pseudotypes impacts transduction efficiency. As controls, we included the surface glycoprotein(s) of well-characterized FLUAV strains, A/WSN/33 (H1N1) (WSN-HA, WSN-NA), A/South Carolina/1/18 (H1N1) (1918-HA), A/Brevig Mission/1/1918 (H1N1) (1918-NA), and A/Singapore/1/57 (H2N2) (H2N2-HA, H2N2-NA), as well as the glycoproteins of Nipah virus (NiV-G, NiV-F) and vesicular stomatitis virus (VSV-G).

**Fig 2:**
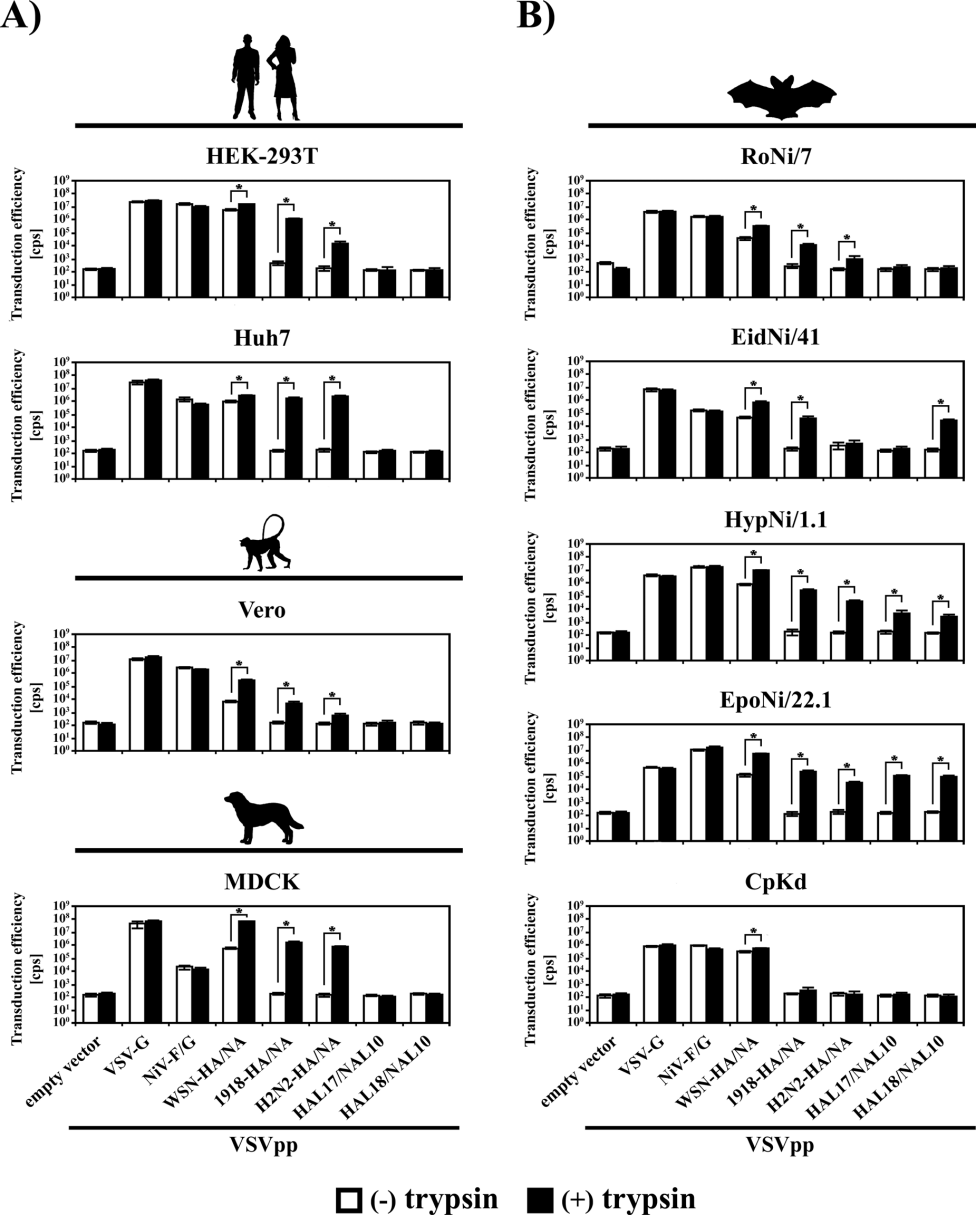
Surface glycoproteins of batFLUAV enable pseudotype entry into different bat but not human, simian and canine cell lines. Vesicular stomatitis virus-based pseudotypes (VSVpp) harboring no or the indicated viral glycoproteins were either left untreated (white bars) or treated with trypsin (black bars), before they were inoculated onto mammalian cell lines of human (HEK-293T, Huh7), simian (Vero) and canine (MDCK) origin (A) or bat cell lines (RoNi/7, EidNi/41, HypNi/1.1, EpoNi/22.1, CpKd) representing five different bat species (B). At 18–20 h post inoculation, the transduction efficiency was measured by quantification of the activity of the VSVpp-encoded luciferase (given as counts per second, cps, on a logarithmic scale). The result of a single representative experiment carried out with quadruplicate samples is shown. Similar results were obtained in four independent experiments carried out with separate pseudotype preparations. Error bars indicate standard deviations. A two-tailed, unpaired student’s t-test was used to test statistical significance (* = p < 0.05).

We found that none of the human, simian and canine cell lines was susceptible to entry driven by batFLUAV surface proteins (Fig 2A). In contrast, pseudotypes bearing VSV-G, NiV-F/G or WSN-HA/NA could readily enter these cells, whereas pseudotypes that harbored 1918- or H2N2-HA/NA required activation by exogenous trypsin for efficient transduction (Fig 2A). These results are in agreement with expectations, since activation of WSN-HA is known to be independent of trypsin [42–44], although viral infectivity can be enhanced by trypsin treatment.

When we focused on bat-derived cell lines, VSV-G, NiV-F/G and WSN-HA/NA again permitted pseudotype entry without prior trypsin treatment, while pseudotypes harboring 1918-HA/NA or H2N2-HA/NA were only able to transduce some of the bat cell lines after incubation with trypsin (Fig 2B). CpKd cells remained refractory to entry mediated by 1918-HA/NA or H2N2-HA/NA but also showed the lowest susceptibility to all other tested pseudotypes. Notably, three bat cell lines (EidNi/41, HypNi/1.1 and EpoNi/22.1) were susceptible to entry of pseudotypes bearing HAL and NAL of batFLUAV (Fig 2B), demonstrating that surface glycoproteins of batFLUAV can mediate cellular entry. Entry into the three bat cell lines was robust (1–3 log units above the threshold) and required prior treatment of pseudotypes with trypsin, which presumably resulted in the proteolytic activation of HAL. Furthermore, pseudotypes bearing HAL17/NAL10 or HAL18/NAL10 were both able to enter HypNi/1.1 and EpoNi/22.1 cells while EidNi/41 cells could only be transduced by pseudotypes bearing HAL18/NAL10 (Fig 2B), suggesting that batFLUAV of the HL17NL10 and HL18NL11 subtype might exhibit subtle differences in entry efficiency or cell tropism. Finally, HAL-proteins with a C-terminal FLAG tag facilitated host cell entry, although with somewhat reduced efficiency as compared to their untagged counterparts, indicating that the proteins used to study HAL expression and virion incorporation (Fig 1) were functional (data not shown). Taken together, we showed that batFLUAV surface proteins can mediate entry into certain bat cell lines. For further studies on the entry process, we focused on EpoNi/22.1 cells since they showed the highest susceptibility to entry driven by batFLUAV surface proteins.

### Expression of batFLUAV-NAL in pseudotype producing cells does not impact particle infectivity

**Fig 3:**
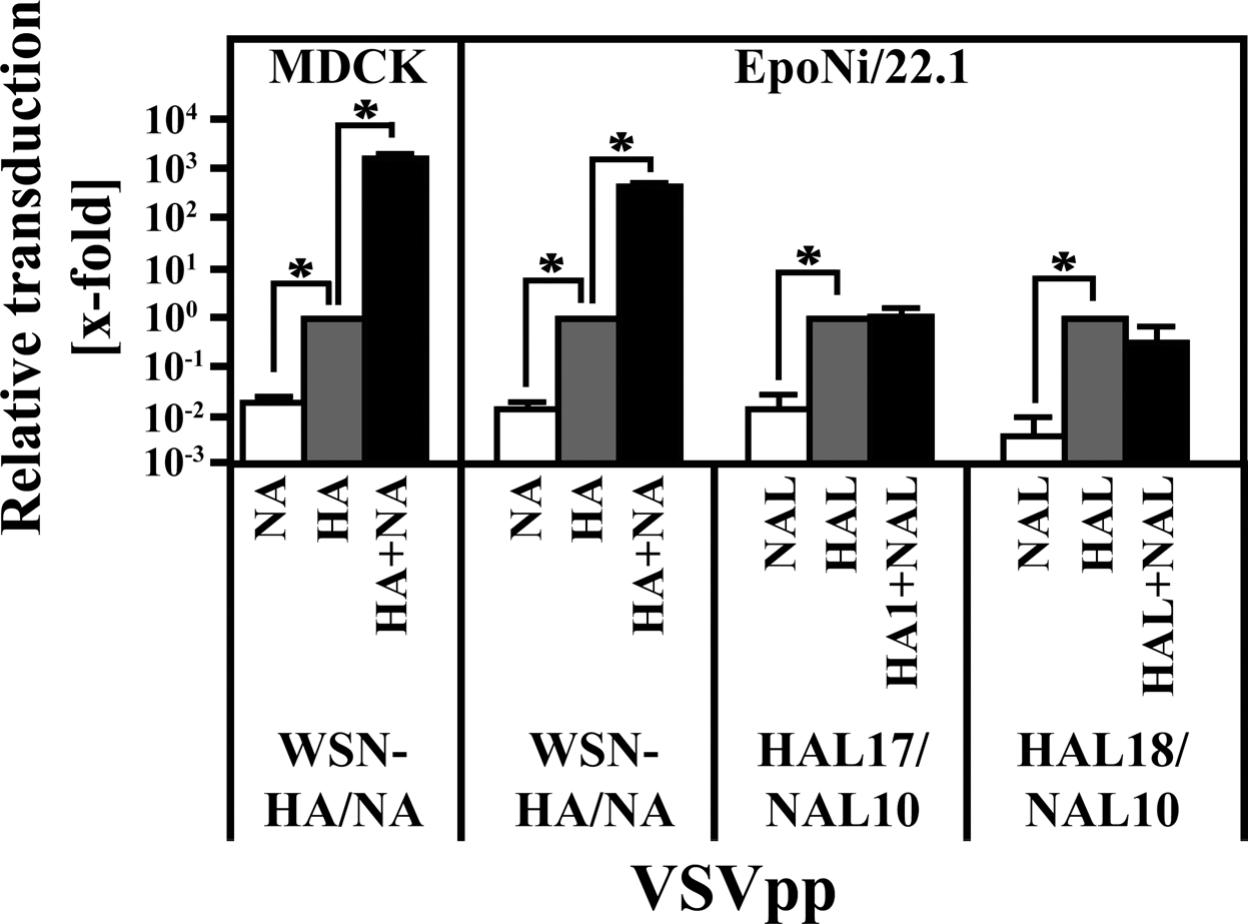
NAL of batFLUAV has no impact on the transduction efficiency of vectors bearing HAL. Vesicular stomatitis virus-based pseudotypes (VSVpp) harboring the indicated surface proteins of FLUAV or batFLUAV were treated with trypsin before inoculation of MDCK and EpoNi/22.1 cells. At 18–20 h post inoculation, the transduction efficiency was measured by quantification of the activity of the VSVpp-encoded luciferase. The combined data from three independent experiments (quadruplicate samples) with separate pseudotype preparations are shown. Transduction efficiencies were normalized against pseudotypes harboring only HA or HAL (set as 1) and are given as x-fold changes on a logarithmic scale. Error bars indicate standard error of the mean. A two-tailed, paired student’s t-test was used to test statistical significance (* = p < 0.05).

The NA proteins of human FLUAV facilitate release of progeny particles from infected cells by removing sialic acids from the cell surface. To study the impact of the batFLUAV-NAL on transduction efficiency, we produced pseudotypes bearing batFLUAV-HAL, -NAL or both proteins. For comparison, we generated pseudotypes harboring WSN-HA, WSN-NA or both proteins. These pseudotypes were then used for inoculation of MDCK (inoculated with pseudotypes bearing WSN proteins) and EpoNi/22.1 (inoculated with pseudotypes bearing WSN or batFLUAV proteins) cells. Pseudotypes harboring only WSN-NA were not able to transduce target cells (Fig 3) while pseudotypes bearing either WSN-HA alone or in combination with WSN-NA transduced both MDCK and EpoNi/22.1, as expected. Transduction efficiency was ~500–1,500-fold higher when WSN-NA was expressed in cells used for pseudotype production, in keeping with the findings that the presence of NA is required for efficient release of HA-bearing vectors and infectious FLUAV [32, 45, 46]. Pseudotypes harboring NAL were not infectious while pseudotypes bearing HAL robustly transduced EpoNi/22.1 cells, indicating that batFLUAV-HAL, like WSN-HA, is sufficient to mediate host cell entry. However, unlike WSN-NA, the expression of NAL in pseudotype producer cells did not increase transduction efficiency of HAL-harboring pseudotypes (Fig 3), suggesting that NAL is not required for release and/or infectivity of HAL containing particles, at least in the experimental system chosen.

### BatFLUAV-HAL does not use sialic acids for host cell entry

**Fig 4:**
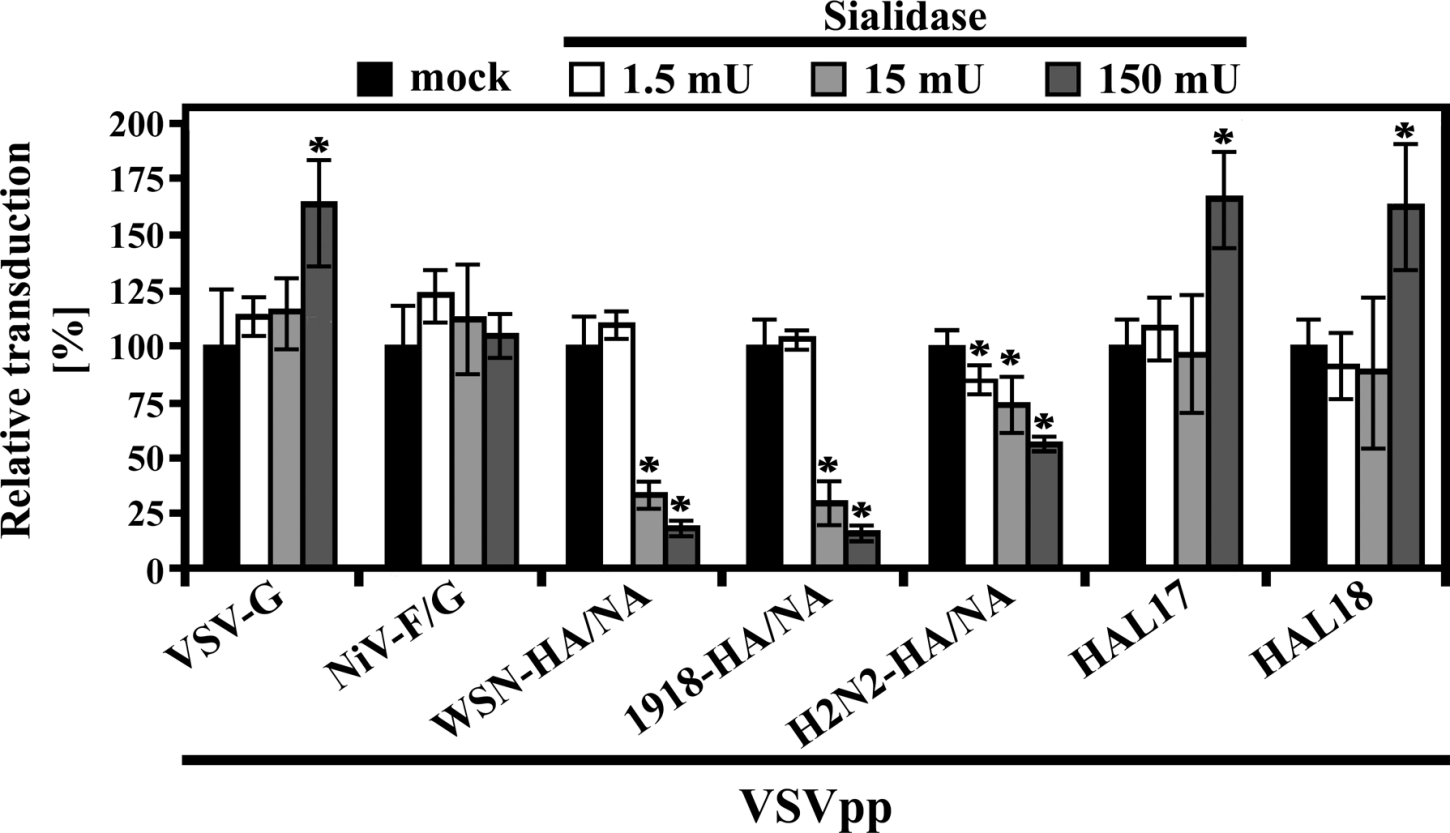
HAL of batFLUAV does not require sialic acids for host cell entry. EpoNi/22.1 cells were incubated for 1.5 h in the absence (black bars) or presence (white and grey bars) of increasing concentrations of exogenous sialidase and were subsequently inoculated with trypsin-treated vesicular stomatitis virus-based pseudotypes (VSVpp) harboring the indicated viral glycoproteins. At 1 h post inoculation, the inoculum was removed, the cells were washed and further incubated for 18–20 h with fresh medium before transduction efficiency was measured by quantification of the activity of VSVpp-encoded luciferase. Transduction of sialidase-treated cells is shown relative to that measured for mock-treated cells (on a linear scale), which was set at 100%. The result of a single representative experiment carried out with quadruplicate samples is shown. Similar results were obtained in two independent experiments carried out with separate pseudotype preparations. Error bars indicate standard deviations. A two-tailed, unpaired student’s t-test was used to test statistical significance of differences measured for sialidase- versus mock-treated samples (* = p < 0.05).

FLUAV employ alpha-2,3- (avian viruses) and alpha-2,6-linked (human viruses) sialic acids as receptors for host cell entry [47–51]. In order to assess the potential role of sialic acids in HAL-driven entry, we pre-treated the cells with escalating doses of bacterial neuraminidase before transduction. Neuraminidase treatment of EpoNi/22.1 cells reduced transduction mediated by the FLUAV-HA proteins, as expected (Fig 4). In contrast, sialidase treatment had no effect on pseudotype entry mediated by batFLUAV-HAL, NiV-F/G or VSV-G. Moreover, pre-treatment of EpoNi/22.1 cells at the highest dose (150 mU) even enhanced transduction driven by HAL and VSV-G (Fig 4). These results indicate that HAL does not use sialic acids for host cell entry and suggest that removal of sialic acids might even increase batFLUAV infectivity, potentially by increasing accessibility of a cellular receptor.

### HAL-driven entry of batFLUAV relies on endosomal acidification

**Fig 5:**
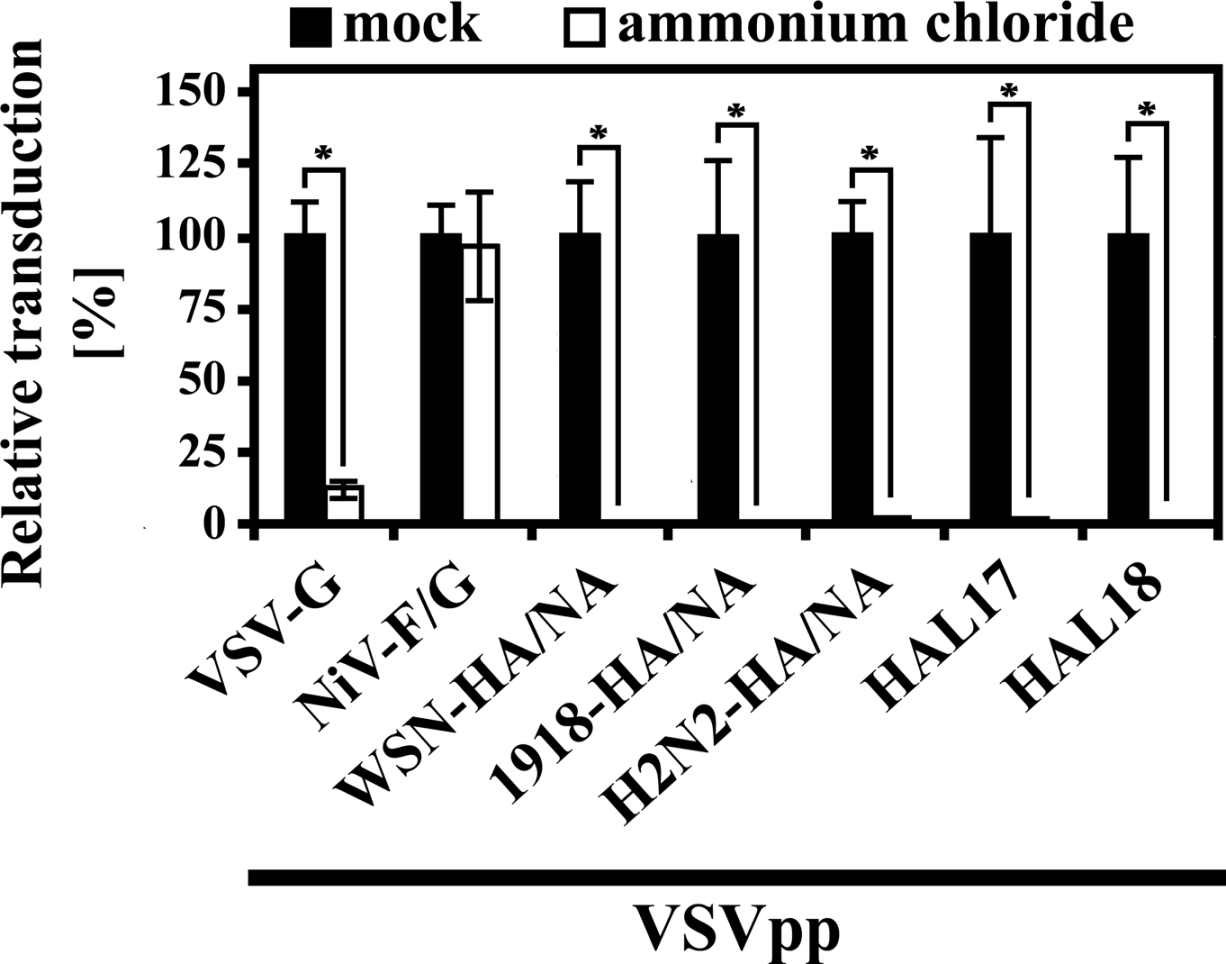
Entry driven by batFLUAV-HAL relies on an acidic pH. EpoNi/22.1 cells incubated for 3 h in the absence (black bars) or presence (white bars) of ammonium chloride (50 mM) were subsequently inoculated with trypsin-treated vesicular stomatitis virus-based pseudotypes (VSVpp) harboring the indicated viral glycoproteins. At 1 h post inoculation, the inoculum was removed and the cells were further incubated for 18–20 h in the presence or absence of ammonium chloride before transduction efficiency was measured by quantification of the activity of VSVpp-encoded luciferase. For each of the different pseudotypes, transduction efficiency (given as percentage on a linear scale) was normalized against the respective control (water). The result of a single representative experiment carried out with quadruplicate samples is shown. Similar results were obtained in two independent experiments carried out with separate pseudotype preparations. Error bars indicate standard deviations. A two-tailed, unpaired student’s t-test was used to test statistical significance (* = p < 0.05).

Endosomal low pH triggers FLUAV-HA for membrane fusion. Therefore, we investigated whether increasing the endosomal pH in EpoNi/22.1 cells by ammonium chloride treatment impacts HAL-driven entry. As expected, ammonium chloride treatment led to a decrease in transduction efficiency mediated by pseudotypes bearing the HA-proteins of FLUAV of the H1N1 and H2N2 subtype and VSV-G (Fig 5). In contrast, pseudotype entry orchestrated by NiV-F and -G was unaffected, again in keeping with published data [52, 53]. Finally, HAL-driven entry was markedly reduced by ammonium chloride, demonstrating that the membrane fusion activity of batFLUAV-HAL is triggered by acidification (Fig 5).

### TMPRSS2 activates batFLUAV-HAL

**Fig 6:**
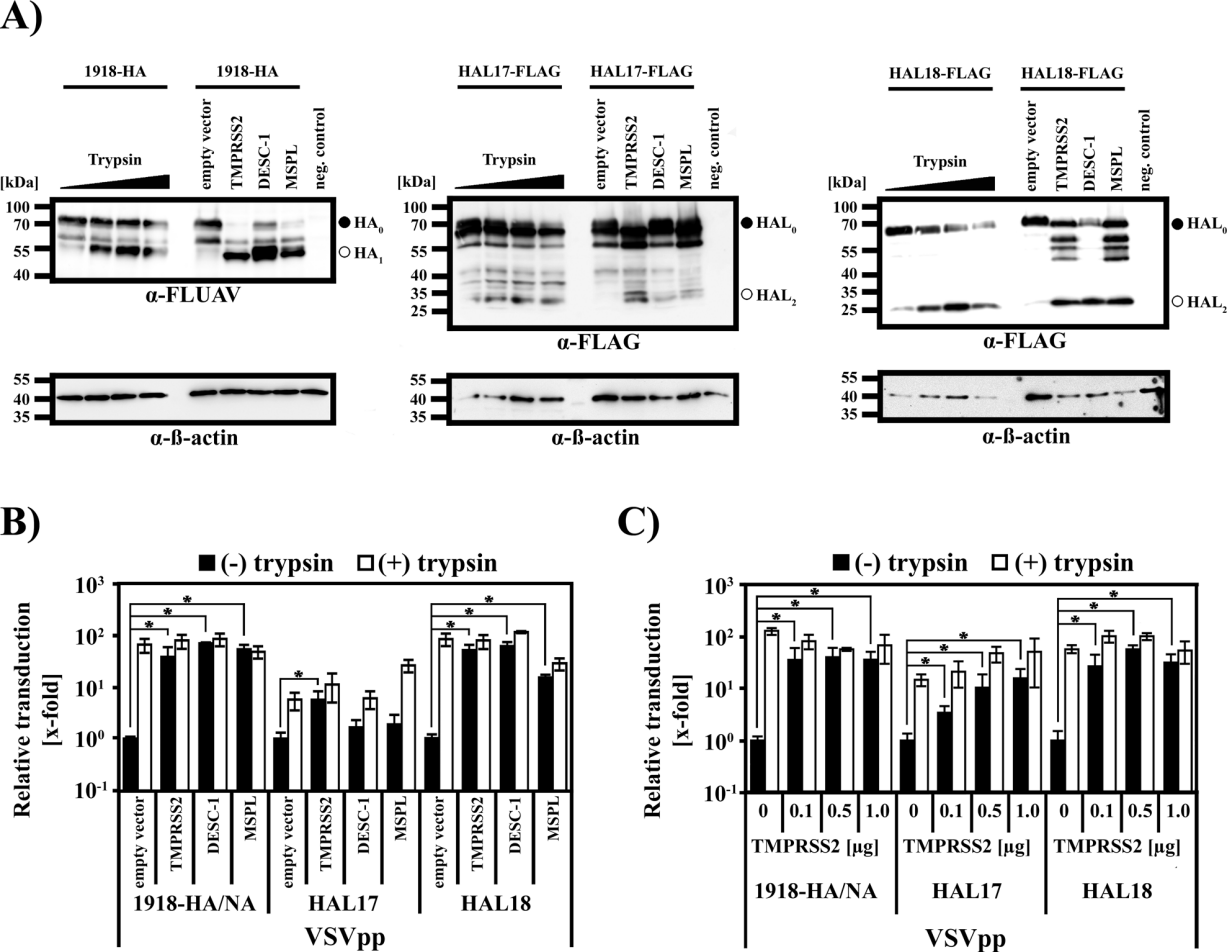
Human proteases that activate FLUAV-HA for cell entry also activate batFLUAV-HAL. (A) HEK-293T cells were transfected with plasmids encoding HA or HAL proteins and either trypsin treated or cotransfected with plasmids encoding type II transmembrane serine proteases. Transfection of empty vector served as negative control. Cleavage of HA/HAL proteins was analyzed by SDS-PAGE and Western blotting, employing antibodies against FLUAV-HA (α-FLUAV) and the FLAG epitope (α-FLAG). Detection of ß-actin served as loading control. Signals corresponding to uncleaved precursor proteins are marked by black circles, while products of proteolytic cleavage are indicated by white circles. The results were confirmed in a separate experiment. To assess proteolytic activation of HA/HAL proteins, vesicular stomatitis virus-based pseudotypes (VSVpp) were produced in cells transfected to express the indicated type II transmembrane serine proteases (B) or different amounts of TMPRSS2 (C). Pseudotypes were either directly used for transduction of EpoNi/22.1 cells (black bars) or previously treated with trypsin (white bars). At 24 h post inoculation, transduction efficiency was measured by quantification of the activity of VSVpp-encoded luciferase in cell lysates. For normalization, transduction by HA- or HAL-bearing pseudotypes that were produced in the absence of type II transmembrane serine protease expression (empty vector) and not treated with trypsin was set as 1. The result of a single representative experiment carried out with quadruplicate samples is presented. Similar results were obtained in three independent experiments carried out with separate pseudotype preparations. Error bars indicate standard deviations. A two-tailed, unpaired student’s t-test was used to test statistical significance (* = p < 0.05).

FLUAV-HA is synthesized as an inactive precursor and requires activation by host cell proteases to be responsive to low pH, the trigger for HA-driven membrane fusion [12–14]. Members of the TTSP family activate FLUAV-HA in cell culture [33, 54–58] and TMPRSS2 was previously shown to be essential for FLUAV-HA activation and viral spread in mice [59]. Therefore, we asked whether TTSPs able to activate FLUAV-HA can also activate batFLUAV-HAL. For this, we first investigated batFLUAV-HAL cleavage by TMPRSS2, DESC-1 and MSPL, and compared it to cleavage by trypsin. Cleavage of the 1918-HA served as positive control. We found that 1918-HA was efficiently processed by all proteases tested, as expected. Moreover, we could show that coexpression of TMPRSS2, DESC-1 and MSPL, and trypsin treatment resulted in cleavage of the HAL precursor (HAL_0_) determined by the appearance of bands corresponding to the HAL_2_ subunit (Fig 6A). While HAL18 was comparably cleaved by all tested TTSPs, HAL17 cleavage by TMPRSS2 was more pronounced than proteolysis by DESC-1 and MSPL (Fig 6A). Moreover, HAL18 was generally more sensitive to cleavage by TTSPs than HAL17 (Fig 6A). In order to assess whether batFLUAV-HAL cleavage by TTSPs also leads to HAL activation for host cell entry, we produced pseudotypes harboring batFLUAV-HAL (HAL17 or HAL18) in the presence of TMPRSS2, DESC-1 and MSPL. As a control, pseudotypes bearing 1918-HA and -NA were included in this experiment. The pseudotypes were treated with trypsin to activate HA/HAL or were mock-treated before addition to EpoNi/22.1 cells. Pseudotypes bearing 1918-HA and -NA and produced in the presence of TMPRSS2, DESC-1 and MSPL or treated with trypsin robustly transduced target cells (Fig 6B). In contrast, infectivity of FLUAV-HA pseudotypes produced in the absence of TTSPs or not treated with trypsin was in the background range (Fig 6B). Similarly, batFLUAV-HAL-bearing pseudotypes were activated by trypsin or TTSPs, including TMPRSS2 (Fig 6B). However, differences in the activation of HAL17 and HAL18 were observed and correlated with the efficiency of HAL protein cleavage, as determined above (Fig 6A). Thus, expression of TMPRSS2 but not DESC-1 and MSPL conferred robust infectivity to HAL17-bearing pseudotypes while all proteases were able to efficiently activate HAL18. Moreover, transfection of escalating amounts of TMPRSS2-encoding plasmids increased infectivity of HAL17-bearing pseudotypes in a concentration-dependent manner. In contrast, transfection of even the lowest amount of TMPRSS2 plasmid was sufficient to confer maximal infectivity of HAL18-bearing pseudotypes, confirming that the efficiency of TMPRSS2-mediated activation of HAL is subtype specific (Fig 6C). In sum, proteolytic activation of batFLUAV-HAL is critical for HAL-driven cell entry and proteases able to activate HA can also activate HAL.

## Discussion

The identification of two new FLUAV, subtypes H17N10 (HL17NL10) and H18N11 (HL18NL11), in new-world bats [16, 17] suggests that bats could serve as a natural reservoir of FLUAV [16, 17]. Unraveling the zoonotic potential of batFLUAV is of great importance since FLUAV are major human pathogens, responsible for influenza epidemics and pandemics. While the batFLUAV replication machinery appears to be functional in different mammalian (including human) cells [16, 18–21, 60], reassortment with FLUAV and FLUBV is unlikely [18, 19]. Regarding batFLUAV tropism of the viral surface proteins, HAL and NAL, only limited information is available, which indicate that batFLUAV do not engage with canonical FLUAV receptors [17, 22–24]. However, until very recently no proof of functional activity of batFLUAV surface proteins was available [17, 22–27]. Here, we employed a vector system to analyze batFLUAV-HAL and -NAL. We show that HAL mediates entry into certain bat cell lines and that entry does not depend on the presence of sialic acids on the cell surface. Moreover, we demonstrate that NAL is not required for production of infectious HAL-bearing particles, at least under the conditions examined. Finally, our studies revealed that trypsin and TTSPs activate HAL for host cell entry.

We used a VSV-based vector system to study cellular entry of HAL and NAL-bearing particles. VSV pseudotypes allow convenient analysis of entry driven by diverse glycoproteins [28, 31, 61], although one should keep in mind that due to differences in particle shape and efficiency of glycoprotein incorporation pseudotypes might not adequately mirror all aspects of cellular entry of authentic viruses [62, 63]. We found that cell lines frequently used for FLUAV propagation were not susceptible to transduction by HAL and NAL bearing particles, which is in agreement with the finding that replacement of batFLUAV-HAL and -NAL by their FLUAV counterparts is required for spread of batFLUAV in the cell lines studied so far [18, 19]. In contrast, inoculation of bat cell lines originating from five different species of micro- and megachiropteran bats revealed that three cell lines, EidNi/41, HypNi/1.1, EpoNi/22.1, were susceptible to entry mediated by batFLUAV surface proteins. EpoNi/22.1 cells showed the highest susceptibility and were thus used for further studies, while EidNi/41 cells were found to be robustly susceptible only to transduction by pseudotypes harboring the HAL18. Collectively, these findings suggest that batFLUAV surface proteins can mediate entry into certain bat cells and that entry efficiency might differ between batFLUAV subtypes. Our finding that certain bat cell lines are susceptible to pseudotypes harboring HAL of batFLUAV are in line with observations very recently documented by Maruyama *et al*. who found that out of a diverse panel of bat cell lines tested, cells from *Miniopterus fuliginosus*, *Miniopterus schreibersii* and *Pteropus giganteus* were susceptible to pH-dependent, HAL-driven entry [64]. A cell line derived from *Eidolon helvum* spleen cells was found to be non-susceptible in contrast to our findings with a kidney cell line established from the same species, suggesting that receptor expression might differ between organs. Somewhat more surprising, Maruyama and colleagues also observed HAL-driven entry into MDCK cells [64], which was not detected in the present study, and these discrepant results might be attributed to use of MDCK cells from different sources or to differences in the method used to quantify pseudotype entry. Finally, it is noteworthy that cell lines from bats inhabiting different geographical locations (Africa, Asia, Europe) were found to be susceptible to HAL-driven entry, suggesting that entry is not a bottleneck for spread of batFLUAV between bat species.

The finding that batFLUAV surface proteins can facilitate entry into bat-derived target cells allowed us to investigate which viral and cellular components contribute to the entry process. We first focused on NAL. The expression of this protein, unlike expression of NA, in pseudotype-producing cells did not increase the titers of vectors harboring the corresponding batFLUAV-HAL protein. However, this finding does not exclude that NAL, like the NA of FLUAV, acts as a receptor-destroying enzyme. Thus, HEK-293T cells used for pseudotype preparation were not susceptible to HAL-driven entry, most likely because they do not express the appropriate receptor. It thus remains to be analyzed whether batFLUAV-NAL increases release of HAL-bearing vectors and authentic batFLUAV from susceptible bat cell lines. These endeavors might be challenging since transfection of bat cell lines by calcium-phosphate precipitation and liposome-based reagents was inefficient (not shown).

The FLUAV-HA is sufficient to mediate viral binding and entry into target cells and our findings indicate that the same applies to batFLUAV-HAL. In contrast to FLUAV-HA, however, HAL does not depend on the presence of sialic acids for entry. Thus, treatment of EpoNi/22.1 cells with sialidase did not decrease HAL-mediated pseudotype entry. These findings are in keeping with the work by Maruyama *et al*. [64] and with structural data indicating that HAL does possess a distorted putative sialic acid binding site [17, 24]. Contrarily, high amounts of sialidase increased entry efficiency, potentially by increasing access to the elusive receptor. In addition, removal of sialic acids might increase electrostatic interactions of batFLUAV-HAL with cell surface factors, since sialic acids are negatively charged. Although HAL-driven entry was independent of sialic acids, it did require endosomal acidification (in accordance with Maruyama *et al*. [64]), which is known to trigger the membrane fusion activity of HA. Most likely, protonation also triggers batFLUAV-HAL for membrane fusion. However, it cannot be disregarded that the inhibitory effect of ammonium chloride was due to blockade of pH-dependent endosomal cysteine proteases, which activate the surface proteins of several coronaviruses and ebolaviruses [65–68].

Cleavage-activation of FLUAV-HA by host cell proteases is essential for FLUAV infectivity. Several TTSPs can cleave and activate HA in cell culture and recent studies demonstrated that TMPRSS2 is essential for FLUAV spread in mice [32, 33, 58, 59]. Moreover, polymorphisms in TMPRSS2 were shown to impact severity of influenza in humans [69]. Treatment of batFLUAV-HAL-expressing cells with trypsin led to proteolytic cleavage of HAL and exposure of HAL-bearing pseudotypes to trypsin was required for efficient transduction of target cells, indicating that proteolytic processing is also required for HAL function. Moreover, coexpression of batFLUAV-HAL with TMPRSS2, DESC-1 or MSPL resulted in proteolytic cleavage of HAL and rendered the particles infectious in the absence of trypsin treatment, suggesting that batFLUAV-HAL can utilize human proteases for their activation. Finally, titration of the amounts of TMPRSS2 had differential effects on the proteolytic cleavage of HAL17 and HAL18 and on infectivity of pseudotypes bearing these proteins, hinting towards subtle differences in the efficiency of TMPRSS2 use by these subtypes. Whether bat TMPRSS2 is also able to cleave and activate batFLUAV-HAL remains to be investigated.

Collectively, our results are most compatible with a scenario in which human cells allow for batFLUAV-HAL activation and triggering but fail to express a receptor, which can be employed by HAL for host cell entry. These results, jointly with the documented observation that the batFLUAV replication machinery is functional in human cells [16, 18, 19] suggest that HAL adaptation to a human receptor might be the major hurdle batFLUAV need to overcome to spread in humans. It will thus be highly interesting to identify the nature of this receptor and its interface with batFLUAV-HAL.

Of note, during the preparation of this manuscript, Maruyama and colleagues published a manuscript reporting batFLUAV-HAL-driven entry into bat cell lines different from those used in the present study (Maruyama *et al*., 2015, doi: 10.1016/j.virol.2015.11.002.). Both studies show that HAL-driven entry requires prior proteolytic HAL-activation by trypsin and endosomal acidification but is independent of sialic acids. The present work extends these findings by demonstrating that HAL can utilize the human enzyme TMPRSS2 for its activation.

## References

[1] WHO. Influenza (Seasonal) Fact sheet No. 211: World Health Organisation; 2014 [updated March 2014; cited 2015 26.11.2015]. Available from: http://www.who.int/mediacentre/factsheets/fs211/en/.

[2] Cox NJ, Subbarao K. Global epidemiology of influenza: past and present. Annual review of medicine. 2000;51:407–21. 10.1146/annurev.med.51.1.407 . 10774473

[3] Parrish CR, Kawaoka Y. The origins of new pandemic viruses: the acquisition of new host ranges by canine parvovirus and influenza A viruses. Annual review of microbiology. 2005;59:553–86. 10.1146/annurev.micro.59.030804.121059 . 16153179

[4] Webster RG, Bean WJ, Gorman OT, Chambers TM, Kawaoka Y. Evolution and ecology of influenza A viruses. Microbiological reviews. 1992;56(1):152–79. 1579108 10.1128/mr.56.1.152-179.1992 PMC372859

[5] Liu D, Shi W, Shi Y, Wang D, Xiao H, Li W, et al Origin and diversity of novel avian influenza A H7N9 viruses causing human infection: phylogenetic, structural, and coalescent analyses. Lancet. 2013;381(9881):1926–32. 10.1016/S0140-6736(13)60938-1 . 23643111

[6] Taubenberger JK, Morens DM. Pandemic influenza—including a risk assessment of H5N1. Revue scientifique et technique. 2009;28(1):187–202. 19618626 10.20506/rst.28.1.1879 PMC2720801

[7] Palese P. Influenza: old and new threats. Nature medicine. 2004;10(12 Suppl):S82–7. 10.1038/nm1141 . 15577936

[8] Taubenberger JK, Morens DM. 1918 Influenza: the mother of all pandemics. Emerging infectious diseases. 2006;12(1):15–22. 10.3201/eid1201.050979 16494711 PMC3291398

[9] Carroll SM, Higa HH, Paulson JC. Different cell-surface receptor determinants of antigenically similar influenza virus hemagglutinins. The Journal of biological chemistry. 1981;256(16):8357–63. . 6167577

[10] Suzuki Y, Matsunaga M, Nagao Y, Taki T, Hirabayashi Y, Matsumoto M. Ganglioside GM1b as an influenza virus receptor. Vaccine. 1985;3(3 Suppl):201–3. . 4060848 10.1016/0264-410x(85)90104-5

[11] Suzuki Y, Kato H, Naeve CW, Webster RG. Single-amino-acid substitution in an antigenic site of influenza virus hemagglutinin can alter the specificity of binding to cell membrane-associated gangliosides. Journal of virology. 1989;63(10):4298–302. 2476569 10.1128/jvi.63.10.4298-4302.1989 PMC251045

[12] Bertram S, Glowacka I, Steffen I, Kuhl A, Pohlmann S. Novel insights into proteolytic cleavage of influenza virus hemagglutinin. Reviews in medical virology. 2010;20(5):298–310. 10.1002/rmv.657 . 20629046 PMC7169116

[13] Bouvier NM, Palese P. The biology of influenza viruses. Vaccine. 2008;26 Suppl 4:D49–53. 19230160 10.1016/j.vaccine.2008.07.039 PMC3074182

[14] Palese P, Shaw ML. Orthomyxoviridae In: Knipe DM, Howley PM, editors. Fields Virology. 2 5th ed: Lippincott Williams & Wilkins; 2007 p. 1647–89.

[15] Fouchier RA, Munster V, Wallensten A, Bestebroer TM, Herfst S, Smith D, et al Characterization of a novel influenza A virus hemagglutinin subtype (H16) obtained from black-headed gulls. Journal of virology. 2005;79(5):2814–22. 10.1128/JVI.79.5.2814-2822.2005 15709000 PMC548452

[16] Tong S, Li Y, Rivailler P, Conrardy C, Castillo DA, Chen LM, et al A distinct lineage of influenza A virus from bats. Proceedings of the National Academy of Sciences of the United States of America. 2012;109(11):4269–74. 10.1073/pnas.1116200109 22371588 PMC3306675

[17] Tong S, Zhu X, Li Y, Shi M, Zhang J, Bourgeois M, et al New world bats harbor diverse influenza A viruses. PLoS pathogens. 2013;9(10):e1003657 10.1371/journal.ppat.1003657 24130481 PMC3794996

[18] Juozapaitis M, Aguiar Moreira E, Mena I, Giese S, Riegger D, Pohlmann A, et al An infectious bat-derived chimeric influenza virus harbouring the entry machinery of an influenza A virus. Nature communications. 2014;5:4448 10.1038/ncomms5448 . 25055345 PMC5533278

[19] Zhou B, Ma J, Liu Q, Bawa B, Wang W, Shabman RS, et al Characterization of uncultivable bat influenza virus using a replicative synthetic virus. PLoS pathogens. 2014;10(10):e1004420 10.1371/journal.ppat.1004420 25275541 PMC4183581

[20] Poole DS, Yu S, Cai Y, Dinis JM, Muller MA, Jordan I, et al Influenza A virus polymerase is a site for adaptive changes during experimental evolution in bat cells. Journal of virology. 2014;88(21):12572–85. 10.1128/JVI.01857-14 25142579 PMC4248895

[21] Turkington HL, Juozapaitis M, Kerry PS, Aydillo T, Ayllon J, Garcia-Sastre A, et al Novel Bat Influenza Virus NS1 Proteins Bind Double-Stranded RNA and Antagonize Host Innate Immunity. Journal of virology. 2015;89(20):10696–701. 10.1128/JVI.01430-15 26246567 PMC4580192

[22] Sun X, Shi Y, Lu X, He J, Gao F, Yan J, et al Bat-derived influenza hemagglutinin H17 does not bind canonical avian or human receptors and most likely uses a unique entry mechanism. Cell reports. 2013;3(3):769–78. 10.1016/j.celrep.2013.01.025 . 23434510

[23] Wu Y, Wu Y, Tefsen B, Shi Y, Gao GF. Bat-derived influenza-like viruses H17N10 and H18N11. Trends in microbiology. 2014;22(4):183–91. 10.1016/j.tim.2014.01.010 . 24582528 PMC7127364

[24] Zhu X, Yu W, McBride R, Li Y, Chen LM, Donis RO, et al Hemagglutinin homologue from H17N10 bat influenza virus exhibits divergent receptor-binding and pH-dependent fusion activities. Proceedings of the National Academy of Sciences of the United States of America. 2013;110(4):1458–63. 10.1073/pnas.1218509110 23297216 PMC3557073

[25] Garcia-Sastre A. The neuraminidase of bat influenza viruses is not a neuraminidase. Proceedings of the National Academy of Sciences of the United States of America. 2012;109(46):18635–6. 10.1073/pnas.1215857109 23100536 PMC3503194

[26] Li Q, Sun X, Li Z, Liu Y, Vavricka CJ, Qi J, et al Structural and functional characterization of neuraminidase-like molecule N10 derived from bat influenza A virus. Proceedings of the National Academy of Sciences of the United States of America. 2012;109(46):18897–902. 10.1073/pnas.1211037109 23012237 PMC3503196

[27] Zhu X, Yang H, Guo Z, Yu W, Carney PJ, Li Y, et al Crystal structures of two subtype N10 neuraminidase-like proteins from bat influenza A viruses reveal a diverged putative active site. Proceedings of the National Academy of Sciences of the United States of America. 2012;109(46):18903–8. 10.1073/pnas.1212579109 23012478 PMC3503178

[28] Hoffmann M, Muller MA, Drexler JF, Glende J, Erdt M, Gutzkow T, et al Differential sensitivity of bat cells to infection by enveloped RNA viruses: coronaviruses, paramyxoviruses, filoviruses, and influenza viruses. PloS one. 2013;8(8):e72942 10.1371/journal.pone.0072942 24023659 PMC3758312

[29] Muller MA, Raj VS, Muth D, Meyer B, Kallies S, Smits SL, et al Human coronavirus EMC does not require the SARS-coronavirus receptor and maintains broad replicative capability in mammalian cell lines. mBio. 2012;3(6). 10.1128/mBio.00515-12 23232719 PMC3520110

[30] Biesold SE, Ritz D, Gloza-Rausch F, Wollny R, Drexler JF, Corman VM, et al Type I interferon reaction to viral infection in interferon-competent, immortalized cell lines from the African fruit bat Eidolon helvum. PloS one. 2011;6(11):e28131 10.1371/journal.pone.0028131 22140523 PMC3227611

[31] Kuhl A, Hoffmann M, Muller MA, Munster VJ, Gnirss K, Kiene M, et al Comparative analysis of Ebola virus glycoprotein interactions with human and bat cells. The Journal of infectious diseases. 2011;204 Suppl 3:S840–9. 10.1093/infdis/jir306 21987760 PMC3189982

[32] Chaipan C, Kobasa D, Bertram S, Glowacka I, Steffen I, Tsegaye TS, et al Proteolytic activation of the 1918 influenza virus hemagglutinin. Journal of virology. 2009;83(7):3200–11. 10.1128/JVI.02205-08 19158246 PMC2655587

[33] Zmora P, Blazejewska P, Moldenhauer AS, Welsch K, Nehlmeier I, Wu Q, et al DESC1 and MSPL activate influenza A viruses and emerging coronaviruses for host cell entry. Journal of virology. 2014;88(20):12087–97. 10.1128/JVI.01427-14 25122802 PMC4178745

[34] Zimmer G, Locher S, Berger Rentsch M, Halbherr SJ. Pseudotyping of vesicular stomatitis virus with the envelope glycoproteins of highly pathogenic avian influenza viruses. The Journal of general virology. 2014;95(Pt 8):1634–9. 10.1099/vir.0.065201-0 . 24814925

[35] Berger Rentsch M, Zimmer G. A vesicular stomatitis virus replicon-based bioassay for the rapid and sensitive determination of multi-species type I interferon. PloS one. 2011;6(10):e25858 10.1371/journal.pone.0025858 21998709 PMC3187809

[36] Kruger N, Hoffmann M, Weis M, Drexler JF, Muller MA, Winter C, et al Surface glycoproteins of an African henipavirus induce syncytium formation in a cell line derived from an African fruit bat, Hypsignathus monstrosus. Journal of virology. 2013;87(24):13889–91. 10.1128/JVI.02458-13 24067951 PMC3838219

[37] Hanika A, Larisch B, Steinmann E, Schwegmann-Wessels C, Herrler G, Zimmer G. Use of influenza C virus glycoprotein HEF for generation of vesicular stomatitis virus pseudotypes. The Journal of general virology. 2005;86(Pt 5):1455–65. 10.1099/vir.0.80788-0 . 15831958

[38] Kruger N, Hoffmann M, Drexler JF, Muller MA, Corman VM, Sauder C, et al Functional properties and genetic relatedness of the fusion and hemagglutinin-neuraminidase proteins of a mumps virus-like bat virus. Journal of virology. 2015;89(8):4539–48. 10.1128/JVI.03693-14 25741010 PMC4442385

[39] Klenk HD, Rott R, Orlich M, Blodorn J. Activation of influenza A viruses by trypsin treatment. Virology. 1975;68(2):426–39. . 173078 10.1016/0042-6822(75)90284-6

[40] Klenk HD, Rott R, Orlich M. Further studies on the activation of influenza virus by proteolytic cleavage of the haemagglutinin. The Journal of general virology. 1977;36(1):151–61. 10.1099/0022-1317-36-1-151 . 142124

[41] Lazarowitz SG, Choppin PW. Enhancement of the infectivity of influenza A and B viruses by proteolytic cleavage of the hemagglutinin polypeptide. Virology. 1975;68(2):440–54. . 128196 10.1016/0042-6822(75)90285-8

[42] Goto H, Kawaoka Y. A novel mechanism for the acquisition of virulence by a human influenza A virus. Proceedings of the National Academy of Sciences of the United States of America. 1998;95(17):10224–8. 9707628 10.1073/pnas.95.17.10224 PMC21489

[43] Goto H, Wells K, Takada A, Kawaoka Y. Plasminogen-binding activity of neuraminidase determines the pathogenicity of influenza A virus. Journal of virology. 2001;75(19):9297–301. 10.1128/JVI.75.19.9297-9301.2001 11533192 PMC114497

[44] Lazarowitz SG, Goldberg AR, Choppin PW. Proteolytic cleavage by plasmin of the HA polypeptide of influenza virus: host cell activation of serum plasminogen. Virology. 1973;56(1):172–80. . 4795670 10.1016/0042-6822(73)90296-1

[45] Air GM, Laver WG. The neuraminidase of influenza virus. Proteins. 1989;6(4):341–56. 10.1002/prot.340060402 . 2482974

[46] Roberts NA. Anti-influenza drugs and neuraminidase inhibitors. Progress in drug research Fortschritte der Arzneimittelforschung Progres des recherches pharmaceutiques. 2001;56:195–237. . 11417114 10.1007/978-3-0348-8319-1_5

[47] Rogers GN, Paulson JC, Daniels RS, Skehel JJ, Wilson IA, Wiley DC. Single amino acid substitutions in influenza haemagglutinin change receptor binding specificity. Nature. 1983;304(5921):76–8. . 6191220 10.1038/304076a0

[48] Nobusawa E, Aoyama T, Kato H, Suzuki Y, Tateno Y, Nakajima K. Comparison of complete amino acid sequences and receptor-binding properties among 13 serotypes of hemagglutinins of influenza A viruses. Virology. 1991;182(2):475–85. . 2024485 10.1016/0042-6822(91)90588-3

[49] Matrosovich M, Tuzikov A, Bovin N, Gambaryan A, Klimov A, Castrucci MR, et al Early alterations of the receptor-binding properties of H1, H2, and H3 avian influenza virus hemagglutinins after their introduction into mammals. Journal of virology. 2000;74(18):8502–12. 10954551 10.1128/jvi.74.18.8502-8512.2000 PMC116362

[50] Gambaryan AS, Tuzikov AB, Piskarev VE, Yamnikova SS, Lvov DK, Robertson JS, et al Specification of receptor-binding phenotypes of influenza virus isolates from different hosts using synthetic sialylglycopolymers: non-egg-adapted human H1 and H3 influenza A and influenza B viruses share a common high binding affinity for 6'-sialyl(N-acetyllactosamine). Virology. 1997;232(2):345–50. 10.1006/viro.1997.8572 . 9191848

[51] Baigent SJ, McCauley JW. Influenza type A in humans, mammals and birds: determinants of virus virulence, host-range and interspecies transmission. BioEssays: news and reviews in molecular, cellular and developmental biology. 2003;25(7):657–71. 10.1002/bies.10303 . 12815721

[52] Diederich S, Moll M, Klenk HD, Maisner A. The nipah virus fusion protein is cleaved within the endosomal compartment. The Journal of biological chemistry. 2005;280(33):29899–903. 10.1074/jbc.M504598200 . 15961384

[53] Diederich S, Sauerhering L, Weis M, Altmeppen H, Schaschke N, Reinheckel T, et al Activation of the Nipah virus fusion protein in MDCK cells is mediated by cathepsin B within the endosome-recycling compartment. Journal of virology. 2012;86(7):3736–45. 10.1128/JVI.06628-11 22278224 PMC3302499

[54] Okumura Y, Takahashi E, Yano M, Ohuchi M, Daidoji T, Nakaya T, et al Novel type II transmembrane serine proteases, MSPL and TMPRSS13, Proteolytically activate membrane fusion activity of the hemagglutinin of highly pathogenic avian influenza viruses and induce their multicycle replication. Journal of virology. 2010;84(10):5089–96. 10.1128/JVI.02605-09 20219906 PMC2863848

[55] Hamilton BS, Gludish DW, Whittaker GR. Cleavage activation of the human-adapted influenza virus subtypes by matriptase reveals both subtype and strain specificities. Journal of virology. 2012;86(19):10579–86. 10.1128/JVI.00306-12 22811538 PMC3457293

[56] Baron J, Tarnow C, Mayoli-Nussle D, Schilling E, Meyer D, Hammami M, et al Matriptase, HAT, and TMPRSS2 activate the hemagglutinin of H9N2 influenza A viruses. Journal of virology. 2013;87(3):1811–20. 10.1128/JVI.02320-12 23192872 PMC3554176

[57] Garten W, Braden C, Arendt A, Peitsch C, Baron J, Lu Y, et al Influenza virus activating host proteases: Identification, localization and inhibitors as potential therapeutics. European journal of cell biology. 2015;94(7–9):375–83. 10.1016/j.ejcb.2015.05.013 . 26095298

[58] Bottcher E, Matrosovich T, Beyerle M, Klenk HD, Garten W, Matrosovich M. Proteolytic activation of influenza viruses by serine proteases TMPRSS2 and HAT from human airway epithelium. Journal of virology. 2006;80(19):9896–8. 10.1128/JVI.01118-06 16973594 PMC1617224

[59] Hatesuer B, Bertram S, Mehnert N, Bahgat MM, Nelson PS, Pohlmann S, et al Tmprss2 is essential for influenza H1N1 virus pathogenesis in mice. PLoS pathogens. 2013;9(12):e1003774 10.1371/journal.ppat.1003774 24348248 PMC3857797

[60] Tefsen B, Lu G, Zhu Y, Haywood J, Zhao L, Deng T, et al The N-terminal domain of PA from bat-derived influenza-like virus H17N10 has endonuclease activity. Journal of virology. 2014;88(4):1935–41. 10.1128/JVI.03270-13 24284327 PMC3911528

[61] Hofmann H, Li X, Zhang X, Liu W, Kuhl A, Kaup F, et al Severe fever with thrombocytopenia virus glycoproteins are targeted by neutralizing antibodies and can use DC-SIGN as a receptor for pH-dependent entry into human and animal cell lines. Journal of virology. 2013;87(8):4384–94. 10.1128/JVI.02628-12 23388721 PMC3624395

[62] Jung C, Le Doux JM. Lentiviruses inefficiently incorporate human parainfluenza type 3 envelope proteins. Biotechnology and bioengineering. 2008;99(4):1016–27. 10.1002/bit.21622 . 17705232

[63] Steffen I, Simmons G. Pseudotyping Viral Vectors With Emerging Virus Envelope Proteins. Current gene therapy. 2016;16(1):47–55. . 26785737 10.2174/1566523216666160119093948

[64] Maruyama J, Nao N, Miyamoto H, Maeda K, Ogawa H, Yoshida R, et al Characterization of the glycoproteins of bat-derived influenza viruses. Virology. 2016;488:43–50. 10.1016/j.virol.2015.11.002 . 26605499 PMC7126434

[65] Chandran K, Sullivan NJ, Felbor U, Whelan SP, Cunningham JM. Endosomal proteolysis of the Ebola virus glycoprotein is necessary for infection. Science. 2005;308(5728):1643–5. 10.1126/science.1110656 . 15831716 PMC4797943

[66] Simmons G, Gosalia DN, Rennekamp AJ, Reeves JD, Diamond SL, Bates P. Inhibitors of cathepsin L prevent severe acute respiratory syndrome coronavirus entry. Proceedings of the National Academy of Sciences of the United States of America. 2005;102(33):11876–81. 10.1073/pnas.0505577102 16081529 PMC1188015

[67] Kawase M, Shirato K, Matsuyama S, Taguchi F. Protease-mediated entry via the endosome of human coronavirus 229E. Journal of virology. 2009;83(2):712–21. 10.1128/JVI.01933-08 18971274 PMC2612384

[68] Qiu Z, Hingley ST, Simmons G, Yu C, Das Sarma J, Bates P, et al Endosomal proteolysis by cathepsins is necessary for murine coronavirus mouse hepatitis virus type 2 spike-mediated entry. Journal of virology. 2006;80(12):5768–76. 10.1128/JVI.00442-06 16731916 PMC1472567

[69] Cheng Z, Zhou J, To KK, Chu H, Li C, Wang D, et al Identification of TMPRSS2 as a Susceptibility Gene for Severe 2009 Pandemic A(H1N1) Influenza and A(H7N9) Influenza. The Journal of infectious diseases. 2015;212(8):1214–21. 10.1093/infdis/jiv246 . 25904605 PMC7107393

